# Genetic Predisposition for Immune System, Hormone, and Metabolic Dysfunction in Myalgic Encephalomyelitis/Chronic Fatigue Syndrome: A Pilot Study

**DOI:** 10.3389/fped.2019.00206

**Published:** 2019-05-24

**Authors:** Melanie Perez, Rajeev Jaundoo, Kelly Hilton, Ana Del Alamo, Kristina Gemayel, Nancy G. Klimas, Travis J. A. Craddock, Lubov Nathanson

**Affiliations:** ^1^Dr. Kiran C. Patel College of Osteopathic Medicine, Nova Southeastern University, Fort Lauderdale, FL, United States; ^2^Department of Psychology and Neuroscience, Nova Southeastern University, Fort Lauderdale, FL, United States; ^3^Institute for Neuro Immune Medicine, Nova Southeastern University, Fort Lauderdale, FL, United States; ^4^Veterans Affairs Medical Center, Miami, FL, United States; ^5^Department of Computer Science, Nova Southeastern University, Fort Lauderdale, FL, United States

**Keywords:** myalgic encephalomyelitis/chronic fatigue syndrome, genome-wide, single-nucleotide polymorphism, immune system, hormone, metabolic

## Abstract

**Introduction:** Myalgic Encephalomyelitis/ Chronic Fatigue Syndrome (ME/CFS) is a multifactorial illness of unknown etiology with considerable social and economic impact. To investigate a putative genetic predisposition to ME/CFS we conducted genome-wide single-nucleotide polymorphism (SNP) analysis to identify possible variants.

**Methods:** 383 ME/CFS participants underwent DNA testing using the commercial company 23andMe. The deidentified genetic data was then filtered to include only non-synonymous and nonsense SNPs from exons and microRNAs, and SNPs close to splice sites. The frequencies of each SNP were calculated within our cohort and compared to frequencies from the Kaviar reference database. Functional annotation of pathway sets containing SNP genes with high frequency in ME/CFS was performed using over-representation analysis via ConsensusPathDB. Furthermore, these SNPs were also scored using the Combined Annotation Dependent Depletion (CADD) algorithm to gauge their deleteriousness.

**Results:** 5693 SNPs were found to have at least 10% frequency in at least one cohort (ME/CFS or reference) and at least two-fold absolute difference for ME/CFS. Functional analysis identified the majority of SNPs as related to immune system, hormone, metabolic, and extracellular matrix organization. CADD scoring identified 517 SNPs in these pathways that are among the 10% most deleteriousness substitutions to the human genome.

## Introduction

Myalgic Encephalomyelitis/Chronic Fatigue Syndrome (ME/CFS) is a complex illness characterized by disabling fatigue, disturbed sleep patterns, pain, and flu-like symptoms. Patients report a high degree of physical disability, and a decreased quality of life with 24% being homebound (1), causing a US economic loss ranging from of $9.1 to $51 billion (2). Currently, there are three main sources of diagnosis criteria, the Center for Disease Control (CDC) Empiric (3), Fukuda (4), and Canadian Consensus (5), showing 2.54, 1.0, and 0.10% of the population affected, respectively. This variation highlights the lack of a concrete illness definition. Although research studies have identified various aspects such as immune abnormalities and exposure to toxins relevant to the pathogenesis of ME/CFS (6), ME/CFS is still not yet fully understood. The genetic and environmental pathogenesis of ME/CFS remains unclear. Currently, treatment of ME/CFS is dependent on management of symptomology and improvement on quality of life (6). An improved understanding of the molecular mechanisms affected and dysfunction in the regulatory systems will translate into better diagnostic methods and more targeted approaches to treatment. There are numerous studies suggesting that genes and single nucleotide polymorphisms (SNPs) within those genes might play a role in the development and progression of ME/CFS (7–9). Results of these studies are very interesting and useful, however, one of these studies was focused on mitochondrial DNA (7), and the other two were limited by 80 study subjects (8, 9). The aim of the current study is to increase the size of the ME/CFS cohort and identify the most harmful variants associated with ME/CFS.

## Materials and Methods

### Patient Population

Individuals with ME/CFS were selected through an online English pre-screening questionnaire via the RedCap platform. The study was restricted to adults (18–70 years of age) that endorsed a clinically diagnoses of chronic fatigue syndrome (CFS), post-infection fatigue (IF), or myalgic encephalomyelitis (ME) and endorsed criteria meeting the 1994 CDC definition of CFS (4): four or more of the following symptoms over a minimum of 6 consecutive months and not predating fatigue: sore throat, tender cervical or axillary lymph nodes, muscle pain, multiple joint pain without swelling or redness, headaches of new type, pattern or severity, unrefreshing sleep, post-exertional malaise, and impaired memory or concentration. Furthermore, study subjects were excluded if they had HIV infection, or dementia precluding full participation/consent. Qualified prescreened participants then completed an online consent form describing the study in detail, asking them to accept or decline the opportunity to continue with the study, via the RedCap online platform. Consenting participants then securely uploaded their genotyping data received from 23andMe into a secure database using the RedCap online platform.

#### Ethics Approval and Consent to Participate

All study subjects signed an informed consent approved by the Institutional Review Board (IRB) of Nova Southeastern University (NSU). Ethics review and approval for data analysis was also obtained by the IRB of NSU.

### 23andME Genotyping

23andME processes saliva containing DNA that was sent by the study subjects collected with the 23andMe kits according to the supplied instructions. The 23andMe CLIA-certified lab extracted DNA and processed the DNA on a genotyping chip that reads hundreds of thousands of variants in the human genome. Samples were collected starting in July 2016 until August 2018 and processed with 23andMe chip versions 4 (~570 k SNPs; prior to August 2017) and version 5 (~640 k SNPs; after August 2017). Genotyping calls were performed by 23andMe. Personalized reports based on well-established scientific and medical research were returned to study subjects and subsequently uploaded to the NSU RedCap online platform.

### SNP Filtering and Analysis

All variants received from study participants were annotated using SeattleSeq 138 (10) for Genes, Distance-To-Nearest Splice Site, and microRNAs. Based on the annotation we focused our analysis on only non-synonymous and non-sense SNPs located in the gene's coding regions, near the splice sites and in microRNAs. The frequency of each of these SNPs was calculated in ME/CFS cohort (study participants). We compared these frequencies with the frequencies of the corresponding SNPs from the reference database Kaviar [hg19 (GRCh37)] (11). Kaviar contains over 162 million SNPs from 35 projects, including dbSNP, 1000Genomes and other and does not include the data from cancer genomes.

For functional analysis we selected SNPs that satisfied following criteria: the frequency at least 10% in either reference or ME/CFS cohort and the ratio in frequencies between the ME/CFS cohort and the reference cohort is more than two in either direction (Supplementary Table 1).

All variants that prevail in ME/CFS cohort were also scored using the Combined Annotation Dependent Depletion algorithm (CADD) (12) (Supplementary Table 2).

### Functional Annotation

Functional annotation of SNPs was performed using the ConsensusPathDB (13–15) to provide biological pathway information. Over-representation analysis (13) incorporating the Kyoto Encyclopedia of Genes and Genomes (KEGG) (73.0) (16), Netpath (1.1.2015) (17), the Integrating Network Objects with Hierarchies (INOH) (1.1.2015) (18), Biocarta (2009_05_12) (19), Humancyc (18.5) (20), Signalink (8.1.2015) (21), Edinburgh human metabolic network (Ehmn) (1.1.2015) (22), Reactome (51) (23), Wikipathways (9.1.2015) (24) and the Pathway Interaction Database (PID) (2014_02_14) (25) pathway sets was used to interpret the functions the identified SNPs may play. Here the significance of the observed overlap between the gene module and the members of known pathways, compared to random expectations, was calculated based on a hypergeometric distribution. A minimum overlap of two genes between the gene module and the pathway set at a *p*-value cutoff of 0.01 was required. Specifically, the *p*-value was calculated as the probability of randomly finding k or more successes from the population in N total draws. Thus, small *p*-values indicate a greater over-representation than expected by chance. As many of the identified pathways share SNP genes the relation between functions was mapped as a network between identified pathway nodes where edges indicate a number of shared genes. These networks were visualized with Cytoscape version 3.3.0 (26). Pathways sharing at least 30% of SNPs were clustered and organized via circular layout, while the remainder were organized via a perfuse force-directed layout based on the number of shared genes.

## Results and Discussion

**Figure 1:**
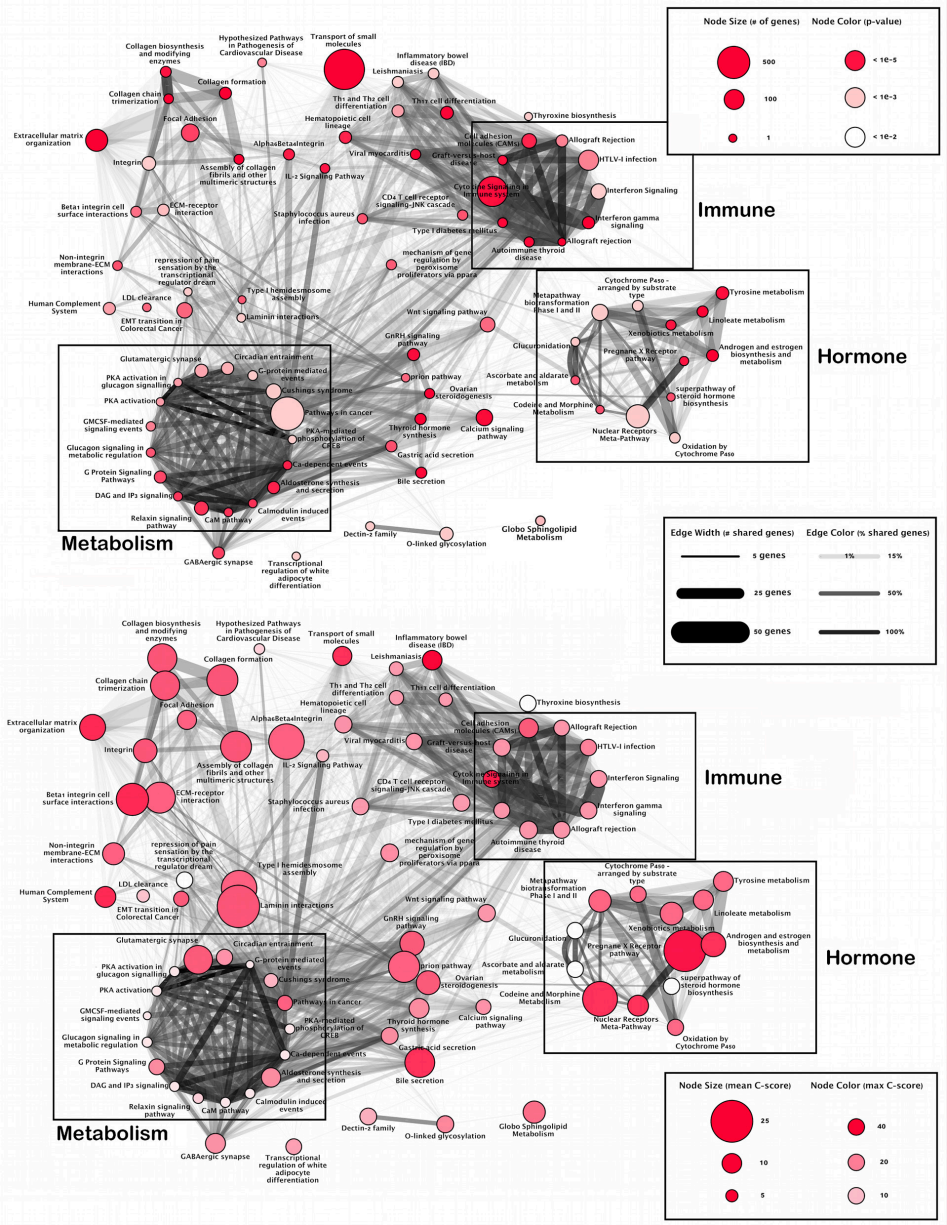
Pathway overlap networks. Pathways identified via over-representation analysis (nodes) connected by shared genes with SNPs (edges). Upper panel gives total number of SNPs per pathway and likelihood of annotation (*p*-value). Lower panel shows pathways most affected by deleterious SNPs.

Functional analysis of SNPs identified three main clusters of pathways as sharing at least 30% SNP related genes (Figure 1). The first is dominated in size via the pathway Cytokine Signaling in Immune System and includes other immune-related pathways such as interferon signaling, autoimmune responses, and T-cell receptor signaling. This cluster highlights a module of immune-related SNPs.

The second cluster is dominated in size via the Nuclear-Receptors Meta-Pathway and includes hormone related pathways such as steroid hormone, estrogen, and androgen biosynthesis, glucuronidation, and the pregnane x receptor pathway. This cluster highlights modules of hormone-related SNPs.

The final cluster is dominated in size by Pathways in Cancer, however, closer inspection shows many metabolic processes such as enzyme reactions (protein kinase A, calcium and calmodulin signaling), and G proteins signaling which regulate metabolic enzymes, which are all involved in the regulation of glycogen, sugar and lipid metabolism. This cluster highlights a module of metabolism-related SNPs.

While there is an overlap between the metabolic and immune modules, the hormone module remains isolated with main connections only formed via Ovarian Steroidogenesis and the Wnt signaling pathway. Finally, there is a group of loosely connected pathways involved in an extracellular matrix organization.

While this organization highlights the interplay between immune, hormone and metabolic activity underlying ME/CFS, overlay of the location of CADD scores illustrates where the most deleterious effects occur (Figure 1; lower panel).

Of the 11,485 SNPs that passed prefiltering according to the annotations (see Methods), 8,593 SNPs had frequency more than 10% in either reference or ME/CFS cohort. Out of them, 5,693 SNPs had a two-fold difference between ME/CFS and the reference cohorts in either direction (Supplementary Table 1).

SNPs that prevailed in ME/CFS cohort were scored using the CADD algorithm (12). According to the CADD algorithm, C-scores above 10 indicate that these SNPs are predicted to be among the 10% most harmful, and C-scores above 20 indicate the 1% most deleterious substitutions (12). Table 1 shows 50 SNPs that are the most frequent in the ME/CFS cohort and have C-scores above 10.

**Table 1:** 50 most frequent deleterious SNPs in ME/CFS cohort compared to reference cohort.

**Gene**	**ID**	**ME/CFS frequency**	**Kaviar frequency**	**Frequency ratio**	**C-score**
GPBAR1	rs199986029	7.73E-01	6.00E-06	1.29E+05	36.00
HLA-C	rs41560916	6.27E-01	1.30E-05	4.82E+04	15.55
BCAM	rs3810141	1.02E-01	6.00E-06	1.70E+04	33.00
AAAS	rs150511103	1.93E-01	1.30E-05	1.49E+04	33.00
FGA	rs146387238	1.93E-01	1.30E-05	1.49E+04	33.00
SLC25A13	rs80338723	1.93E-01	1.30E-05	1.49E+04	32.00
MYBPC3	rs112738974	1.93E-01	1.90E-05	1.02E+04	34.00
PEX6	rs112298166	1.93E-01	1.90E-05	1.02E+04	26.80
CYP2D6	rs1135830	4.54E-01	9.70E-05	4.68E+03	24.30
HLA-DRB1	rs112796209	4.15E-01	1.09E-04	3.81E+03	26.10
PLA2G4D	rs147516345	1.59E-01	1.03E-04	1.55E+03	25.60
CYP2A6	rs5031017	3.86E-01	2.64E-04	1.46E+03	24.20
CYP2D6	rs199535154	9.43E-01	2.31E-03	4.08E+02	22.10
DDX51	rs201101053	1.59E-01	7.08E-04	2.25E+02	49.00
LHB	rs34349826	7.42E-01	6.44E-03	1.15E+02	13.18
HLA-A	rs1137110	1.38E-01	2.49E-03	5.57E+01	16.35
HLA-DRB1	rs1136756	4.39E-01	1.00E-02	4.38E+01	14.71
HLA-DRB1	rs9269744	4.05E-01	1.30E-02	3.12E+01	23.80
TPTE	rs1810540	3.45E-01	1.16E-02	2.97E+01	35.00
HLA-DQA1	rs1061172	1.57E-01	1.33E-02	1.18E+01	15.33
C6orf183	rs399561	6.32E-01	6.46E-02	9.78E+00	15.17
C14orf37	rs3829765	8.15E-01	9.75E-02	8.36E+00	15.58
EFCAB4B	rs11062745	2.79E-01	3.39E-02	8.25E+00	21.60
PLD5	rs2810008	5.54E-01	6.71E-02	8.25E+00	16.00
MUC19	rs11564109	2.40E-01	2.95E-02	8.15E+00	24.70
ARHGAP42	rs17647207	1.44E-01	1.82E-02	7.91E+00	23.30
ADAMTS19	rs30645	7.65E-01	9.75E-02	7.85E+00	18.74
LINC01171	rs11605546	2.30E-01	2.97E-02	7.73E+00	15.31
ANKDD1B	rs34358	8.33E-01	1.09E-01	7.65E+00	45.00
ZBED5	rs2232919	1.20E-01	1.61E-02	7.45E+00	24.20
CTC-441N14.4	rs9112	6.03E-01	8.44E-02	7.15E+00	21.70
SLC35B2	rs3187	1.31E-01	1.85E-02	7.07E+00	11.89
PRSS41	rs61747737	1.15E-01	1.63E-02	7.06E+00	13.70
OTOG	rs12422210	2.64E-01	3.76E-02	7.01E+00	15.38
MTCH2	rs1064608	4.57E-01	6.58E-02	6.95E+00	25.00
SULF1	rs6990375	5.12E-01	7.49E-02	6.83E+00	14.77
OTOG	rs11024333	2.95E-01	4.34E-02	6.80E+00	10.26
ART3	rs14773	4.33E-01	6.41E-02	6.76E+00	14.51
PPHLN1	rs12658	3.63E-01	5.45E-02	6.66E+00	15.95
PRICKLE1	rs12658	3.63E-01	5.45E-02	6.66E+00	15.95
VARS2	rs2249464	7.47E-01	1.14E-01	6.56E+00	16.14
MORN2	rs3099950	2.19E-01	3.37E-02	6.50E+00	25.50
AC007956.1	rs2270424	3.68E-01	5.99E-02	6.15E+00	33.00
AREL1	rs2270424	3.68E-01	5.99E-02	6.15E+00	33.00
PRRT4	rs359642	9.50E-01	1.55E-01	6.12E+00	10.83
HUS1	rs2307252	1.67E-01	2.76E-02	6.05E+00	12.72
PRSS56	rs1550094	9.22E-01	1.62E-01	5.68E+00	16.32
C5orf52	rs10051838	2.40E-01	4.35E-02	5.52E+00	17.68
ZNHIT1	rs17319250	4.05E-01	7.41E-02	5.46E+00	10.74
CPLX2	rs3822674	7.05E-01	1.29E-01	5.46E+00	10.05

Of the 50 most frequent deleterious SNPs found in our ME/CFS cohort compared to the reference database (Table 1), 10 were found to have a frequency of 70% or more in the ME/CFS group. This includes *CYP2D6, PRRT4*, and *PRSS56* at a frequency over 90%, *C14orf37, ANKDD1B*, at over 80%, and *GPBAR1, LHB, ADAMTS19, VARS2*, and *CPLX2* at over 70%.

*CYP2D6* (Cytochrome P450 2D6) is primarily expressed in the liver, but also highly expressed in areas of the central nervous system, including the substantia nigra, and is one of the most important enzymes involved in the metabolism of xenobiotics in the body. A significantly higher frequency of polymorphisms *CYP2D6* was found in ME/CFS study subjects with Fibromyalgia than in controls and could differentiate these study subjects s from study subjects with multiple chemical sensitivity (27). *CYP2D6* was found in the xenobiotics metabolism, androgen and estrogen biosynthesis and metabolism, tyrosine metabolism, codeine and morphine metabolism, oxidation by cytochrome P450, metapathway biotransformation phase I and II, and cytochrome P450—arranged by substrate type pathways all of which belong to the hormone related cluster.

*PRSS56* (putative serine protease 56) is a serine protease that has been implicated in human eye development (28) and in the regulation of cerebellum activity of mice in exercise (29). It was not found to be a member of any of the annotated pathways.

*GPBAR1* (G Protein-Coupled Bile Acid Receptor) functions as a cell surface receptor for bile acids and participates in the production of intracellular cAMP and activation of a MAP kinase signaling pathway. This receptor plays a big role in the suppression of macrophage functions and regulation of energy homeostasis by bile acids (30). Finding of the deleterious SNP in GPBAR1 (Table 1) is in agreement with the results of the recent study that showed disturbances in bile acid metabolism in ME/CFS study subjects (31). *GPBAR1* was not among any of the pathways annotated.

*LHB* (luteinizing hormone beta polypeptide) is expressed in the pituitary gland and is essential for spermatogenesis and ovulation by stimulating the testes and ovaries to synthesize steroids (32, 33). *LHB* was found among the GnRH signaling pathway and ovarian steroidogenesis pathway.

*ADAMTS19* is a member of the large *ADAMTS* (a desintegrin-like and metalloprotease with thrombospondin type 1 motif) family of metalloproteases (metal binding enzymes). ADAM proteins are responsible for the proteolytic cleavage of many transmembrane proteins and the release of their extracellular domain. *ADAMTS19* is considered as a possible candidate for premature ovarian failure (34). Only the O-linked glycosylation pathway was found to contain ADAMTS19.

*VARS2* (valyl-tRNA synthetase 2, mitochondrial) is important for the mitochondrial protein synthesis. Mutations in this gene are associated with cardiomyopathy (35), microcephaly and epilepsy (36), deficiency of the mitochondrial respiratory chain complex I and oxidative phosphorylation deficiency (37). *VARS2* was not found among any of the annotated pathways.

*CPLX2* gene encodes the complexin 2 protein that participates in neurotransmitter release by directly interacting with the neuronal SNARE complex (38). *CPLX2* is known to be overexpressed in aging and downregulated by sleep deprivation (39), and this shows a connection of *CPLX2* expression to fatigue. *CPLX2* was also not found among any of the annotated pathways.

The remaining genes *PRRT4, C14orf37*, and *ANKDD1B* are obscure without much literature to support their function and not found among any of the annotated pathways. It was determined that *PRRT4* (proline-rich transmembrane protein 4) showed biased expression in adult ovary, lung, adrenals, CNS and whole brain, while *C14orf37* showed bias in brain, kidney, and ovary (ncbi.nlm.nih.gov). Little information was found for *ANKDD1B* (ankyrin repeat and death domain containing 1B).

Although SNPs in *MYBPC3* and *HLA* genes have lower frequencies in ME/CFS cohort (0.19 for *MYBPC3* and 0.13-0.44 for various *HLA* isoforms, respectively), these SNPs could be used for subgrouping of ME/CFS study subjects in larger studies because of their possible association with ME/CFS and fatigue. Multiple deleterious SNPs in *HLA* genes are in agreement with known impairment of the immune system in ME/CFS (40). Increased frequency of *HLA-DQA1* alleles and decreased expression of *HLA-DRB1* was found to be associated with ME/CFS (41). *MYBPC3* (myosin binding protein C, cardiac) dysfunction is also associated with hypertrophic cardiomyopathy and corresponding fatigue (42).

These results contrast with previous SNP studies in ME/CFS (9, 43–45) which have found statistically significant associations in multiple loci including in neuroendocrine effector and receptor genes (43), TRP ion channels and AChRs (44, 45), and genes regulating the HPA axis (9, 46). This difference is most likely due to a combination of factors such as, (i) differences in array types used between studies, (ii) difference in the methods of analysis, (iii) differences between cohorts and the general heterogeneity of ME/CFS, and (iv) small-effect variants due to the relatively small sample sizes in each of these previous studies, compared to our relatively large cohort.

To date, this is the largest study known using SNP data and its affected pathways in combination with study subjects' self-reported symptoms. The results generated from our study will enhance the current understanding of ME/CFS and will generate new studies, all of which will lead to a better method for diagnosis and targeted genetic therapy. Replicative larger studies are warranted to improve the reliability of the results.

While these results are novel there are some limitations to the current analysis that are worth noting. First, there is no control over the chip version used by 23andMe for genotyping. This can result in loss of precision in the determination of study subject genotyping signature. Second, this initial pilot analysis was only conducted on SNPs in protein coding regions, miRNA regions, and regions close to splice junctions. SNPs in non-coding regions may be important in the cause of the illness. Finally, this analysis does not include rare variants. Moving forward, future studies based on this on-going collection of study subject information will address these limitations, will increase sample size, and provide more detailed statistical analyses. Building on this dataset we also aim to correlate these findings with our on-going research on gene expression (47), miRNA expression and DNA methylation (48).

## Ethics Statement

All subjects signed an informed consent approved by the Institutional Review Board of Nova Southeastern University. Ethics review and approval for data analysis was also obtained by the IRB of Nova Southeastern University.

## Author Contributions

NK, LN, and TC conceived the study. MP, RJ, TC, and LN analyzed data. NK consulted on ME/CFS symptoms. AD, KH, KG, and MP led the recruitment of study participants.

### Conflict of Interest Statement

The authors declare that the research was conducted in the absence of any commercial or financial relationships that could be construed as a potential conflict of interest.
